# Tisdale-score-based risk stratification of QTc prolongations in hospitalized patients receiving azole antifungal therapy—a retrospective study

**DOI:** 10.3389/fcvm.2026.1685152

**Published:** 2026-03-09

**Authors:** Julian Steinbrech, Ute Amann, Michael Irlbeck, Sebastian Clauß, Dorothea Strobach

**Affiliations:** 1 Hospital Pharmacy, LMU University Hospital, Munich, Germany; 2 Doctoral Program Clinical Pharmacy, LMU University Hospital, Munich, Germany; 3 Faculty of Medicine, LMU, Munich, Germany; 4 Department of Anesthesiology, LMU University Hospital, Munich, Germany; 5 Department of Cardiology, LMU University Hospital, Munich, Germany; 6 DZHK (German Center for Cardiovascular Research), Munich Heart Alliance, Munich, Germany; 7 Institute of Surgical Research at the Walter-Brendel-Center of Experimental Medicine, LMU University Hospital, Munich, Germany; 8 Member of the European Reference Network for Rare, low prevalence and complex Diseases of the Heart (ERN GUARD-Heart), Munich, Germany; 9 Interfaculty Center for Endocrine and Cardiovascular Disease Network Modelling and Clinical Transfer (ICONLMU), LMU, Munich, Germany

**Keywords:** antifungal agents, critical care, long QT syndrome, pharmaceutical care, risk assessment

## Abstract

**Background:**

QTc prolongation can trigger potentially lethal arrhythmias. Almost all azole antifungals, which are used in vulnerable patients, prolong the QTc interval and thus, may increase arrhythmia risk. The Tisdale-risk-score allows to identify patients at risk for drug-induced QTc prolongation but has not yet been investigated in this patient population.

**Objective:**

To evaluate the sensitivity and specificity of the Tisdale-score with regard to detected QTc prolongations in patients prescribed systemic azole antifungals.

**Methods:**

For six months (12/23-05/24), prescriptions of systemic azole antifungals were retrospectively recorded in adult inpatients of all medical specialties of a university hospital. Risk factors for QTc prolongation, including concomitant drugs and ECGs, were documented and the Tisdale-score and its sensitivity and specificity were calculated.

**Results:**

In the study period, 319 systemic azole prescriptions (cases) were recorded for 259 patients. The median age of all cases was 61 years, 45% (143) were female. Including the systemic azole, a prescription of ≥2 QT-drugs was present in 283 (89%) cases. The median Tisdale-score was 7 (moderate risk). ECGs after azole initiation were available in 149 cases. Out of these, relevant QTc prolongations occurred in 7 cases (4.7%). Sensitivity of the Tisdale-score was 100%, specificity 30%.

**Conclusion:**

Patients prescribed systemic azole antifungals are at risk of QTc prolongation due to regular use of multiple QT-drugs. However, relevant QTc prolongations were rare in the patient population studied. The Tisdale-score achieved a good sensitivity for the identification of patients at risk for QTc prolongation.

## Introduction

Prolongation of the QTc-interval can result in potentially lethal torsade de pointes (TdP) arrhythmia and is often associated with the intake of QTc-prolonging drugs (1, 2). In addition to drugs, various risk factors for QTc prolongation have been identified including age, sex, comorbidities (cardiac and non-cardiac) and electrolyte disturbances (in particular potassium, magnesium) (1–3).

QTc prolongations occur frequently in hospitalized patients (4–6). ECG monitoring is therefore recommended in medical guidelines, e.g., when additional QTc prolonging drugs are prescribed (7). However, in clinical practice, a risk of QTc prolongation is often overlooked and ECG monitoring is rarely performed (8–11).

Azole antifungals are drugs used systemically for the treatment or prophylaxis of potentially life threatening invasive fungal infections (12, 13). With the exception of isavuconazole, which is reported to shorten the QTc interval, all azole antifungals are associated with QTc prolongations and TdP (14–16). In addition, azoles are high-risk drugs regarding drug-drug interactions (DDI) including elevated serum levels of additional QT-drugs (14, 17). As azoles are often used in very vulnerable patients, e.g., immunocompromised patients due to hemato-oncological diseases or organ transplantation and in intensive care, patients receiving systemic azole antifungals seem to be subject to a particular risk of QTc prolongation (5, 6, 10).

In order to increase awareness of the risk for QTc prolongations, warnings regarding DDI of QTc-prolonging drugs by clinical decision support systems are very frequent in clinical practice. However, over-alerting can lead to alert fatigue and patient-specific factors other than drugs play a relevant role in the genesis of QTc prolongations. Thus, there is a need for patient specific risk stratification if QTc prolonging medication is taken. Various risk scores for QTc prolongation have been published thus far (18–23). However, there is limited data on the transferability and universality of most risk scores, apart from the original patient cohort. The Tisdale-score is a risk score originally developed in intensive care cardiology patients (18). However, the score was already evaluated in a number of further patient cohorts and has shown consistent results regarding the correct identification of patients at risk of QTc prolongation (10, 11, 24). In a study investigating hemato-oncology patients receiving systemic antifungal therapy, the Tisdale-score was found to be the most suitable for use in clinical practice regarding performance and usability compared to four additional QTc risk scores (10). The Tisdale-score has not yet been investigated in patients receiving systemic azole antifungal therapy across medical specialties thus far. The aim of this study therefore was to investigate the sensitivity and specificity of the Tisdale-score with regard to detected QTc prolongations in patients of a university hospital prescribed systemic azole antifungals across all medical specialties.

## Materials and methods

### Study design

This retrospective study investigated patients receiving systemic azole antifungal therapy in a six-month period (December 2023 to May 2024) at the LMU university hospital Munich, campus Großhadern, Germany.

We included patients ≥18 years receiving a systemic azole antifungal and usual pharmaceutical care when prescribed an azole antifungal during hospital stay. The usual care consists of a daily screening of patients prescribed an azole antifungal through the electronic medication softwares Meona (Mesalvo GmbH, Freiburg, Germany) (normal wards) and Q-Care (Health Information Management GmbH, Bad Homburg, Germany) (intensive care wards). For the identified patients, the Tisdale-score (Table 1) is calculated and the calculated risk as well as three ECG recommendations (day 1; days 2–7 of hospitalized azole treatment) communicated to the ward physicians via electronic chart entry or telephone. This usual care is performed for all patients of all medical specialties excluding patients on pulmonology normal wards as these patients are usually patients post lung transplant, hospitalized for short, routine checkups, who receive the azole antifungal as a long-term antifungal prophylaxis.

**Table 1:** Tisdale-score (18).

Parameter	Weight
Age ≥68 years	1
Female	1
Acute myocardial infarction	2
Heart failure	3
Sepsis	3
Potassium ≤3.5 mmol/L	2
Admission QTc ≥450 ms	2
Loop diuretics	1
1 QTc-prolonging drug	3
≥2 QTc-prolonging drugs	3
<7: Low risk	
7–10: Moderate risk	
≥11: High risk	

The study was conducted according to the guidelines of the Declaration of Helsinki, and approved by the Ethics Committee of the LMU hospital Munich (24-0967, 11 December 2024).

### Data collection and evaluation

Data were collected retrospectively. Clinical and laboratory data were obtained from the electronic patient information system (SAP i.s.h.med, Cerner Corporation, North Kansas City, USA). This included demographical data, information on the hospital stay, and relevant diagnoses for QTc prolongation. Relevant laboratory values and QTc-prolonging drugs according to the CredibleMeds classification were collected for day one (laboratory values, QTc-prolonging drugs) and seven (QTc-prolonging drugs) of in-hospital azole treatment. Missing data were scored as normal value. This represents the common method used in the validation of QTc risk scores (10, 22). ECGs and QTc intervals (Bazett 25) were collected up to 7 days prior to (baseline ECG) and 10 days after (follow up ECG) the first azole intake, if available. QTc intervals of ECGs with an automatically calculated QTc ≥480 ms were manually measured by a cardiologist with expertise in rhythmology to reduce falsely measured QTc prolongations due to artefacts or other abnormalities. For cases with more than one follow up ECG available, the ECG with the longest QTc interval was selected for data evaluation. QTc prolongation was defined as a QTc interval ≥500 ms in a follow up ECG or a *Δ*QTc to a prior ECG of ≥60 ms. For patients with a baseline QTc of ≥500 ms, QTc prolongation was defined as a *Δ*QTc of ≥60 ms of a follow up ECG to the baseline QTc. For patients admitted more than once during the study period, each case was documented and evaluated separately. Sensitivity, specificity, positive (PPV) and negative (NPV) predictive value, as well as the area under the receiver operating characteristic curve (ROC) of the Tisdale-score was determined regarding the prediction of QTc prolongation in a follow up ECG. A moderate and high Tisdale-score value was defined as “positive” for QTc prolongation. Quantitative data are presented as median and interquartile range (IQR) or range and qualitative data using their frequency distribution.

## Results

In the study period, 319 systemic azole prescriptions (cases) were recorded for 259 patients. Demographic data and risk factors, as well as the calculated Tisdale-score are shown in Table 2.

**Table 2:** Demographic data and risk factors for QTc prolongations of the study cohort (percentages in relation to number of cases).

Variable	Total (*n* = 319)
Demographic data	
Cases (*n*)	319
Individual patients (*n*)	259
Age [years] [median (IQR)]	61 (52–70)
Age ≥68 years [*n* (%)]	96 (30.1)
Female [*n* (%)]	143 (44.8)
Duration of stay [days] [median (IQR)]	27 (11–45.5)
Normal ward [*n* (%)]	249 (78.1)
Hematology/oncology [*n* (%)]	127 (39.8)
General surgery [*n* (%)]	30 (9.4)
Radiotherapy [*n* (%)]	30 (9.4)
Gastroenterology [*n* (%)]	21 (6.6)
Urology [*n* (%)]	11 (3.4)
Gynaecology [*n* (%)]	10 (3.1)
Neurology [*n* (%)]	6 (1.9)
Cardiac surgery [*n* (%)]	4 (1.3)
Neurosurgery [*n* (%)]	4 (1.3)
Orthopedic surgery [*n* (%)]	4 (1.3)
ENT [*n* (%)]	1 (0.3)
Cardiology [*n* (%)]	1 (0.3)
Intensive care ward [*n* (%)]	70 (21.9)
Anesthesiology [*n* (%)]	43 (61.4)
Internal medicine [*n* (%)]	10 (14.3)
General surgery [*n* (%)]	7 (10.0)
Cardiology/cardiac surgery [*n* (%)]	5 (7.1)
Bone marrow transplant unit [*n* (%)]	3 (4.3)
Neurosurgery [*n* (%)]	2 (2.9)
Death during hospital stay [*n* (%)]	27 (8.5)
Death caused by arrhythmia [*n* (%)]	0 (0)
Relevant diagnoses	
Acute myocardial infarction [*n* (%)]	1 (0.3)
Heart failure [*n* (%)]	18 (5.6)
Congenital long QT syndrome [*n* (%)]	0 (0)
History of syncope [*n* (%)]	5 (1.6)
Sepsis [*n* (%)]	7 (2.2)
Relevant laboratory values	
eGFR (CKD-EPI) < 60 ml/min/1.73^2^ [*n* (%)]	82 (25.7)
Normal ward [n (%)]	61 (24.5)
Intensive care ward [n (%)]	21 (30.0)
Serum potassium ≤3.5 mmol/l [*n* (%)]^a^	33 (10.3)
MELD-Na [median (IQR)]^b^	6 (6–9)

CKD-EPI, chronic kidney disease epidemiology collaboration; eGFR, estimated glomerular filtration rate; ENT, ear, nose, and throat; IQR, interquartile range; MELD-Na, model of end stage liver disease (including sodium).

^a^ Serum potassium not available for 4 cases (1.3%).

^b^ MELD-Na not calculated due to missing data for 46 cases (14.4%).

Based on all cases, the median age was 61 years (range 20-89), 44.8% were female. In 79 (24.8%) cases the azole prescription already existed before hospital admission and was continued during the hospital stay. 27 (8.5%) of the cases died during the study period, of which 4 causes of death could be attributed to a mainly cardiological cause, but not arrhythmias. TdP or other ventricular arrhythmias could not be identified as a cause of death in any of the investigated cases. Relevant diagnoses enhancing the risk of QTc prolongation did not occur frequently in the studied patient cohort. Hypokalemia at azole-initiation was present in 10.3% of cases.

Prescribed QTc prolonging drugs were further evaluated regarding their frequency and classification according to CredibleMeds (Table 3).

**Table 3:** QTc-prolonging drugs in the study cohort (*n* = 319). Number of QTc-prolonging drugs per case and five most prescribed drugs per CredibleMeds category (14).

Variable	Total (*n* = 319)
QTc-prolonging drugs [median (IQR)]	3 (2–4)
Prescription of ≥1 QTc-prolonging drug [*n* (%)]	318 (99.7)
Prescription of ≥2 QTc-prolonging drugs [*n* (%)]	283 (88.7)
Prescription of ≥1 drug with Known Risk of TdP [*n* (%)]	173 (54.2)
Prescribed drugs per case [median (IQR)]	1 (0–1)
Fluconazole [*n* (%)]	117 (36.7)
Propofol [*n* (%)]	23 (7.2)
Azithromycin [*n* (%)]	17 (5.3)
Escitalopram [*n* (%)]	12 (3.8)
Ciprofloxacin/citalopram/erythromycin [n (%) each]	6 (1.9)
Prescription of ≥1 drug with Possible Risk of TdP [*n* (%)]	111 (34.8)
Prescribed drugs per case [median (IQR)]	0 (0–1)
Tacrolimus [*n* (%)]	45 (14.1)
Mirtazapine [*n* (%)]	31 (9.7)
Granisetron [*n* (%)]	24 (7.5)
Dexmedetomidine/melperone [*n* (%)]	4 (1.3)
Gilteritinib/pipamperone/venlafaxine/tizanidine [*n* (%) each]	3 (0.9)
Prescription of ≥1 drug with Conditional Risk of TdP [*n* (%)]	290 (90.9)
Prescibed drugs per case [median (IQR)]	2 (1–3)
Pantoprazole [*n* (%)]	204 (63.9)
Posaconazole [*n* (%)]	108 (33.9)
Piperacillin/Tazobactam [*n* (%)]	69 (21.6)
Torasemide [*n* (%)]	61 (19.1)
Voriconazole [*n* (%)]	48 (15.0)
Prescription of loop diuretic [*n* (%)]	70 (21.9)

IQR, interquartile range; loop diuretics, furosemide, torasemide; TdP, torsade de pointes.

Including azole antifungals, a median of 3 (range 0-9) QTc-prolonging drugs were prescribed at the in-hospital start of azole treatment. A prescription of ≥2 QTc-prolonging drugs was present in 283 (88.7%) cases, in 173 (54.2%) cases QTc-prolonging drugs of the highest risk category according to CredibleMeds (Known Risk of TdP) were prescribed. With regard to QTc-prolonging drugs, which are QT-prolonging regardless of the presence of other conditions (CredibleMeds risk category Known or Possible Risk of TdP), more QTc-prolonging drugs were prescribed on day 7 of inpatient azole treatment compared to day one in 9.1% (29) of cases and less in 12.2% (39).

**Figure 1:**
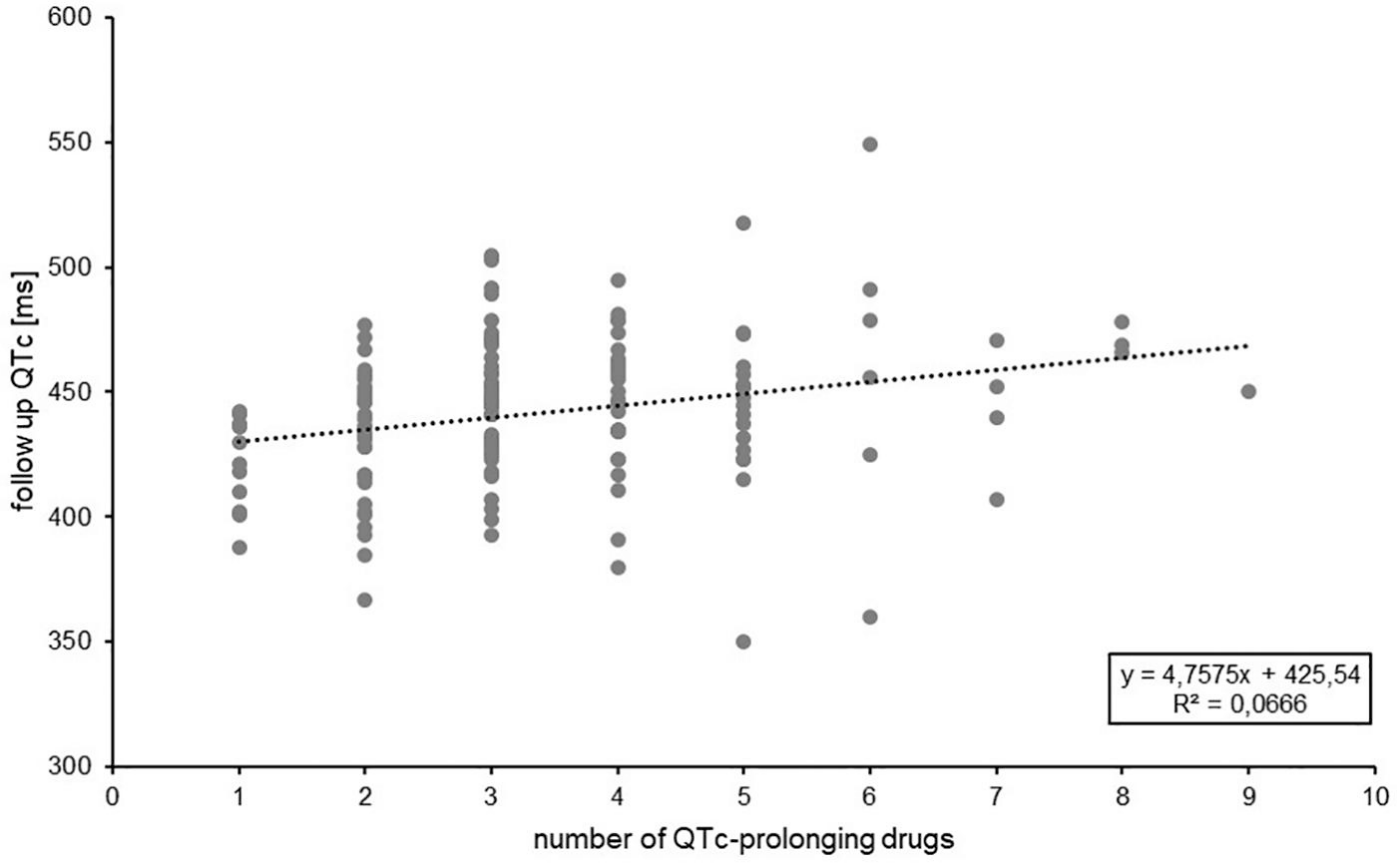
Length of QTc interval [ms] in relation to the number of QTc-prolonging drugs prescribed (*n* = 149 cases).

In Figure 1, the length of the maximum QTc interval of the cases in which a follow-up ECG was available (see Table 4) is plotted against the number of prescribed QTc-prolonging drugs.

**Table 4:** ECG data and Tisdale-score (*n* = 319).

Variable	Total (*n* = 319)
Baseline ECG available (during 7 days prior to azole prescription) [*n* (%)]	112 (35.1)
Baseline QTc ≥450 ms [*n* (%)]	43 (38.4)
Follow up ECG available (during 10 days after first azole prescription) [*n* (%)]	149 (46.7)
Number of follow up ECG per case [median (IQR)]	0 (0–1)
QTc prolongation in follow up ECG (automatic calculation) [*n* (%)]	13 (8.7)
QTc prolongation in follow up ECG (manual measurement) [*n* (%)]	7 (4.7)
Occurrence of TdP [*n* (%)]	0 (0)
Tisdale-score [median (IQR)]	7 (6–8)
High risk [*n* (%)]	23 (7.2)
Moderate risk [*n* (%)]	192 (60.2)
Low Risk [*n* (%)]	104 (32.6)
Tisdale-score for cases with QTc prolongation (*n* = 7)	
High Risk [*n* (%)]	1 (14.3)
Moderate Risk [*n* (%)]	6 (85.7)
Sensitivity (%)	100.0
Specificity (%)	29.6
Positive predictive value (%)	6.5
Negative predictive value (%)	100.0
AUROC (95% CI)	0.74 (0.59–0.90)

IQR, interquartile range; AUROC, area under the receiver operating characteristic curve; TdP, torsade de pointes.

A linear trend towards a prolongation of the QTc interval per prescribed QTc-prolonging drug can be observed, although the regression coefficient was low.

ECG data and calculated performance parameters of the Tisdale-score for the evaluated patient cohort are shown in Table 4.

**Figure 2:**
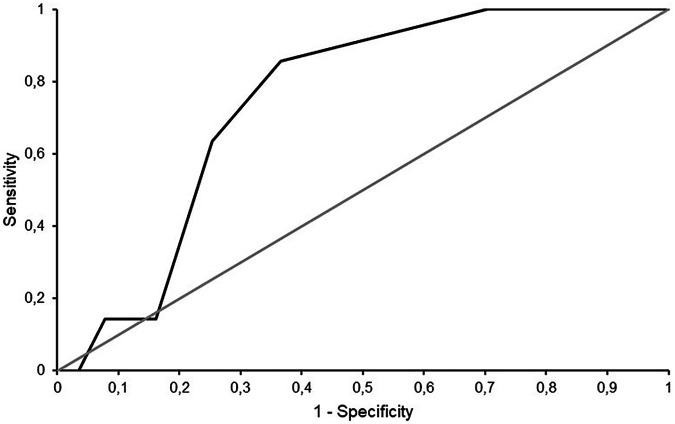
Receiver operating characteristic (ROC) curve of the Tisdale-score.

Clinically relevant QTc prolongation occurred in seven cases (4.7%) in which an ECG was recorded after azole initiation (*n* = 149). In five cases with QTc prolongation according to automatic calculation, manual measurement did not confirm a relevant QTc prolongation. The Tisdale-score provided excellent sensitivity (100.0%) and NPV (100.0%) regarding the correct prediction of QTc prolongations in the investigated patient cohort, however, specificity and PPV were low (29.6% and 6.5%, respectively). The area under the ROC curve was 0.74 (95% CI 0.59–0.90) (Figure 2).

As QTc prolongations occurred in only seven cases, statistical evaluation of potentially relevant risk factors in this patient cohort was not possible. Patient characteristics, relevant risk factors for QTc prolongation are shown in Table 5 for the seven cases, in which a QTc prolongation was detected in a follow-up-ECG.

**Table 5:** Risk factors for QTc prolongation in cases where a QTc prolongation was detected.

Case number	Ward type	Risk factors for QTc prolongation
1	Normal ward (hematology/oncology)	KR: 0, PR: 0, CR: 4, hypokalemia, Tisdale-score: 8
2	ICU (anesthesiology)	Female, KR: 1, PR: 1, CR: 3, Tisdale-score: 8
3	Normal ward (hematology/oncology)	Age ≥68 years, KR: 1, PR: 0, CR: 2, hypokalemia, Tisdale-score: 8
4	ICU (anesthesiology)	Female, KR: 1, PR: 1, CR: 2, baseline QTc ≥450 ms, Tisdale-score: 9
5	ICU (cardiology)	Age ≥68 years, acute myocardial infarction, KR: 2, PR: 0, CR: 2, baseline QTc ≥450 ms, Tisdale-score: 11, deceased (cause of death: hypoxic brain injury after myocardial infarction)
6	ICU (general surgery)	Female, Age ≥68 years, KR: 3, PR: 0, CR: 3, fluconazole dose not adjusted to impaired renal function, Tisdale-score: 9
7	Normal ward (general surgery)	Female, KR: 1, PR: 1; CR: 3, Tisdale-score: 7

CR, number of conditional risk of TdP drugs; ICU, intensive care unit; KR, number of known risk of TdP drugs; PR, number of possible risk of TdP drugs.

Of cases with QTc prolongation, 1 patient died (cause of death: hypoxic brain injury after myocardial infarction). Patients were sufficiently monitored, receiving a median of four (range 1-8) follow up ECGs. Multiple risk factors for QTc prolongation and multiple QTc-prolonging drugs were present in all cases and all cases were correctly identified by the Tisdale-score as patients at risk (moderate/high risk) for QTc prolongation.

## Discussion

This study investigated the use of the Tisdale-score for risk stratification of QTc prolongations in hospitalized patients receiving azole antifungal treatment across medical specialties. The aim was to evaluate the sensitivity and specificity of the Tisdale-score with regard to detected QTc prolongations. All cases in which QTc prolongation was detected in a follow up ECG after azole initiation were correctly classified as moderate or high risk by the Tisdale-score, resulting in a sensitivity of 100% in this patient cohort. Good sensitivities of the Tisdale-score were also achieved in other studies investigating the Tisdale-score in different patient cohorts, reporting sensitivities of 74%–86 (10, 11, 18). The specificity of the Tisdale-score was low in our patient cohort (30%). In comparison, the Tisdale-score achieved a specificity of 77% in the original patient cohort (cardiac intensive care patients). However, other studies in different patient cohorts found similarly low specificities of 22% (hemato-oncology patients receiving systemic antifungal therapy) and 30% (cardiology and gastroenterology patients) (10, 11). Low specificity may lead to patients being incorrectly classified as at risk, resulting in a residual risk of over-alerting. However, due to easy practicability and relatively low cost of ECG recording, patients incorrectly classified as at risk can easily be identified in clinical practice. Additional risk scores have been developed to predict QTc prolongation, with some differing substantially from the Tisdale-score in terms of content and structure. However, in the only study to our knowledge that compared five different risk scores for QTc prolongation in the same patient cohort, the Tisdale-score achieved good predictive results compared to other risk scores, while being easy to calculate, and generated easily interpretable results, leading to the selection of the Tisdale-score for the implementation in this study (10). The results of this study further support the Tisdale-score as a useful tool for clinical practice. Further studies evaluating the use of the Tisdale-score in other patient cohorts could include multivariate logistic regression analyses to investigate the influence of additional risk factors for QTc prolongation in a specific patient cohort and potentially modify the Tisdale-score to achieve better predictive results.

Regarding risk factors for QTc prolongation in this patient cohort, approximately one third of the cases were aged ≥68 years, 10% showed hypokalemia at azole treatment initiation. Renal function was impaired in approximately one fourth of cases, with the proportion being slightly higher in intensive care cases than on normal wards. However, diagnoses contributing to the risk of QTc prolongation according to the Tisdale-score occurred only rarely in this patient cohort. To an extent, this is to be expected, as the diagnoses of acute myocardial infarction and heart failure in particular are likely to be treated mainly on cardiology wards, whereas patients across all medical specialties were investigated in this study. In other studies investigating patients on cardiology wards, a higher prevalence of these diagnoses was observed (11, 18).

Patients prescribed systemic azole antifungals are at risk for drug-induced QTc prolongation due to regular use of multiple QT-drugs. According to the CredibleMeds classification, 54% of cases were prescribed drugs of the highest risk category (“Known Risk of TdP”) (14). Nevertheless, additional QTc-prolonging drugs were prescribed during azole treatment in almost 10% of cases, indicating the widespread use of QTc-prolonging drugs even in patients already at risk for QTc prolongation. Furthermore, an increase in the duration of the QTc interval was observed with an increase in the number of QTc prolonging drugs prescribed, emphasizing the importance of the role of drugs in the development of QTc prolongations. Therefore, these patients could benefit from additional pharmaceutical care. This could include evaluation of therapy alternatives with a lower risk of QTc prolongation, management of DDI and careful dose adjustments of QTc prolonging drugs in impaired renal or hepatic function. This approach should be investigated in further studies.

ECG monitoring (follow up ECG) was performed in 47% of the cases studied. Although this proportion could be further improved, ECG monitoring for almost half of the cases represents a substantial increase compared to other studies investigating the Tisdale-score. In non-cardiology wards, where ECG monitoring recommendations are not part of usual care, as described in this study, follow up ECGs were available in 21% of gastroenterology patients or 24% in hemato-oncology patients receiving systemic antifungal therapy (10, 11). Monitoring recommendations as part of pharmaceutical care therefore have the potential to raise awareness of QTc prolongation and improve the implementation of ECG monitoring in patients at risk.

In the patient cohort investigated, relevant QTc prolongations occurred in only 7 cases (5% of cases with a follow up ECG). The prevalence of relevant QTc prolongation varies between patient cohorts. In intensive care patients, QTc prolongation occurs in 24-28% of patients (2, 5, 6). In hospitalized geriatric patients, QTc prolongations occurred in 34% (4). In contrast, in a study investigating the prevalence of QTc prolongation in hospitalized patients across medical specialties, QTc prolongations occurred in only 0.7% (26). Several reasons may account for the considerable difference in observed QTc prolongations: First, a difference in the occurrence of risk factors for QTc prolongation in different patient cohorts and medical specialties. In this study, cardiac diagnoses contributing to the risk of QTc prolongation occurred in only a few cases, possibly leading to a lower overall risk of QTc prolongation in this cohort. Another reason could be a difference in QTc interpretation. In clinical practice, automatically measured QTc intervals are often used for convenience (27, 28). Since automatically determined QTc intervals are often inaccurate, manual measurement is generally preferred (28, 29). However, other studies have shown that non-cardiology physicians in particular often tend to misinterpret manual QTc measurement (30, 31). To account for possible overestimation by the automatic calculation, we manually measured the QTc intervals of ECGs with an automatically measured QTc interval ≥480 ms and found 5 cases with an incorrectly automatically calculated QTc prolongation. Lastly, differences in the definition of QTc prolongation could have a drastic impact on the reported prevalence of QTc prolongation. In this study, we used a strict definition to include only clinically relevant QTc prolongations. Other studies and even guidelines such as the International Council for Harmonisation E14 guideline suggest cut-off values for the definition of QTc prolongation as low as 450 ms (32). The above mentioned factors could explain the substantially different prevalences of QTc prolongations in different patient cohorts and the low prevalence of QTc prolongations in our cohort. Further studies to investigate the prevalence of clinically relevant QTc prolongations in hospitalized patients and a harmonized definition of clinically relevant QTc prolongation to ensure comparability in clinical research are essential.

Limitations of this study include the limited cohort size and the retrospective nature of the study. Statistical comparison of the risk factors of patients with follow up ECG with QTc prolongation with patients without QTc prolongation was not possible due to the small number of QTc prolongations, the possible causes of which are discussed in detail above. New risk factors or risk factors responsible for the QTc prolongations in the investigated cohort could therefore not be identified. One limitation of using the Bazett formula for QT correction is its known undercorrection at fast heart rates and overcorrection at slow heart rates (33). However, Bazett QT correction is still considered the clinical standard and is used by ECG devices in our hospital.

## Conclusion

In this study, we investigated the use of the Tisdale-score in patients receiving azole antifungal therapy across medical specialties to identify patients at risk for QTc prolongation. In the investigated patient cohort, QTc-prolonging drugs were frequently prescribed, however, QTc prolongations rarely occurred. Due to identification of all patients developing a QTc prolongation, ease of calculation and interpretation, the Tisdale-score is a useful screening tool for risk stratification of QTc prolongations in this patient cohort in clinical practice.

## Data Availability

The raw data supporting the conclusions of this article will be made available by the authors, without undue reservation.
